# Contributions of replicative and translesion DNA polymerases to mutagenic bypass of canonical and atypical UV photoproducts

**DOI:** 10.1038/s41467-023-38255-5

**Published:** 2023-05-04

**Authors:** Brittany N. Vandenberg, Marian F. Laughery, Cameron Cordero, Dalton Plummer, Debra Mitchell, Jordan Kreyenhagen, Fatimah Albaqshi, Alexander J. Brown, Piotr A. Mieczkowski, John J. Wyrick, Steven A. Roberts

**Affiliations:** 1 School of Molecular Biosciences and Center for Reproductive Biology, Washington State University, Pullman, WA 99164 USA; 2 Department of Genetics, Lineberger Comprehensive Cancer Center, University of North Carolina, Chapel Hill, NC 27599 USA

**Keywords:** Genomic instability, Skin cancer

## Abstract

UV exposure induces a mutation signature of C > T substitutions at dipyrimidines in skin cancers. We recently identified additional UV-induced AC > TT and A > T substitutions that could respectively cause *BRAF* V600K and V600E oncogenic mutations. The mutagenic bypass mechanism past these atypical lesions, however, is unknown. Here, we whole genome sequenced UV-irradiated yeast and used reversion reporters to delineate the roles of replicative and translesion DNA polymerases in mutagenic bypass of UV-lesions. Our data indicates that yeast DNA polymerase eta (pol η) has varied impact on UV-induced mutations: protecting against C > T substitutions, promoting T > C and AC > TT substitutions, and not impacting A > T substitutions. Surprisingly, deletion *rad30*Δ increased novel UV-induced C > A substitutions at CA dinucleotides. In contrast, DNA polymerases zeta (pol ζ) and epsilon (pol ε) participated in AC > TT and A > T mutations. These results uncover lesion-specific accurate and mutagenic bypass of UV lesions, which likely contribute to key driver mutations in melanoma.

## Abstract (web summary)

Vandenberg et al. identify differing roles of yeast DNA polymerases during accurate and mutagenic synthesis past common and rare ultraviolet light photoproducts. Similar mechanisms may contribute to driver mutations causing skin cancer in humans.

## Introduction

UV radiation is a potent mutagen that primarily forms cyclobutane pyrimidine dimers (CPDs) and 6-4 photoproducts (6-4PPs) at dipyrimidine sequences in DNA^1^. These bulky lesions can be recognized and repaired by nucleotide excision repair (NER)^2^. However, failure to remove these lesions prior to DNA replication results in single and tandem C > T substitutions in dipyrimidine contexts (i.e., TC, CT, or CC), which constitute the canonical UV mutation signature^3–5^ and highlight why UV exposure is a major risk factor for the development of skin cancers^6^. While typical UV signature mutations comprise nearly 90% of substitutions in skin cancers such as cutaneous melanomas^7^, other mutation types are also observed. In fact, many driver mutations that promote melanomagenesis do not fit the typical UV signature^8,9^. Greater than 50% of melanomas contain a mutation in the *BRAF* gene, which encodes a protein kinase involved in RAS/MAPK signaling that is important for cellular proliferation^10^. Most mutations within this gene occur at amino acid V600. The *BRAF* V600K mutation, which is seen in 5-10% of all sequenced melanoma genomes^11,12^, is caused by a non-dipyrimidine AC > TT substitution^10^. Likewise, *BRAF* V600E is caused by a A > T substitution that also occurs in a non-dipyrimidine context^6^. These mutations, along with greater than 50% of all putative driver mutations in melanoma^9^, do not fit the UV mutation signature. Thus, either non-UV sources of mutation are responsible for the formation of melanoma driver mutations or the currently defined UV mutation signature is incomplete^8^.

Recently, we conducted whole genome sequencing of UV-irradiated yeast cells and observed frequent non-canonical UV-induced mutations, some of which matched the substitutions observed in melanoma driver mutations^8^. In addition to canonical UV signature mutations, UV exposure also induced A > T mutations and AC > TT tandem mutations, whose abundance and transcriptional asymmetry was increased in the absence of global genomic (GG)-NER, indicating they are caused by UV photoproducts^8^. Biochemical and genome-wide damage mapping experiments indicate that UV-induced A > T substitutions are primarily caused by atypical thymine-adenine (TA) photoproducts, which are estimated to form ~50 to 100-fold less abundantly compared to CPDs^8,13^ An AC photoproduct that would be potentially causative of AC > TT substitutions has yet to be identified or biochemically characterized.

UV-induced lesions cause mutations when the NER pathway fails to correct the lesions, allowing them to persist into DNA replication^2^ where error-prone trans-lesion synthesis (TLS) DNA polymerases undergo mutagenic bypass of the lesions. Dysregulation of GG-NER increases UV-induced mutations in a variety of cell types and in the human condition, xeroderma pigmentosum (XP), a disease caused by GG-NER deficiency. XP patients are highly sensitive to UV radiation and individuals are about 10,000 times more likely to develop skin cancer^14^. Along with defects in core global genomic NER factors (i.e. XPA, XPC, etc.)^14^, loss of function of the TLS polymerase, pol η, results in the XPV subgroup of xeroderma pigmentosum. Pol η can bypass TT-containing CPDs in a non-mutagenic manner and lowers the frequency of the canonical C > T UV-induced sustitutions^15^. This is thought to explain why deficiency in pol η causes xeroderma pigmentosum, because there is a much higher level of C > T mutations^16^, resulting from the usage of more error-prone TLS polymerases, like pol ζ^17^. While pol η is protective against the most common C > T substitution, it is mutagenic at TT containing 6-4 PPs, causing T > C substitutions^18^. This indicates that, while protective at some lesions, pol η likely contributes to aspects of the UV mutation signature in cancer. However, the roles of pol η and/or other DNA polymerases in causing non-canonical UV-induced mutations at atypical UV lesions are unknown.

Here, we compare the mutation spectra acquired in the genomes of WT and *rad30*Δ yeast following serial exposure to UVB and UVC irradiation to determine the role of pol η in establishing mutations associated with the atypical UV photoproducts. We found that while yeast pol η was protective against canonical UV-induced C > T substitutions, the polymerase contributed to UV-induced T > C and AC > TT substitutions. Surprisingly, *rad30*Δ had no impact on A > T mutations, indicating that pol η does not participate in the bypass of TA photoproducts, but protected against UV-induced C > A substitutions. Transcriptional asymmetry favoring the transcribed strand suggests C > A mutations are caused by a lesion formed at guanines, which is repaired by transcription coupled (TC)-NER in the absence of pol η. UVB and UVC light produced similar mutation spectra for *rad30*Δ yeast, indicating that the types of mutagenic UV-induced lesions are shared among the medium and short UV wavelengths. Utilization of single-stranded reversion reporters for A > T and AC > TT substitutions recapitulated results from whole genome sequencing, indicating that pol η was not involved in bypass of the TA photoproduct, but contributed to AC > TT mutagenesis. Both UV-induced A > T and AC > TT mutations were completely dependent on the activity of pol ζ, highlighting the requirement of this polymerase to extend synthesis past UV-induced lesions. While pol ζ may also contribute to the insertion of erroneous bases across from both TA and AC photoproducts, utilization of nucleotide selectivity mutants of DNA pol α, δ, and ε^19–21^ suggests that pol ε may have a similar role in this capacity. These results detail the highly coordinated manner in which multiple polymerases contribute to the establishment of UV-induced mutations, including the non-canonical mutations observed in melanoma driver genes.

## Results

### Pol η completes non-mutagenic bypass across the DNA lesion responsible for UV-induced AC > TT mutations

**Fig. 1:**
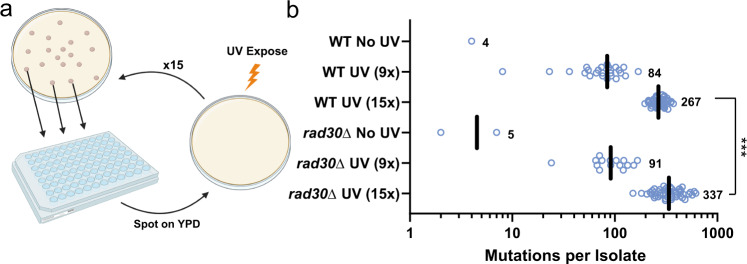
Mutations from whole genome sequencing of UVC irradiated yeast. **a** Experimental procedure for genomic sequencing of UV-induced mutations accrued following 15 exposures to UVC light (25 J/m^2^) in independent yeast isolates. Independent isolates were picked and resuspended at 1 × 10^7^ cells/mL and subsequently spotted on a YPD plate. The plates were exposed to 25 J/m^2^ UVC and incubated at 30 °C for 1–2 days. This was repeated 15× prior to whole genome sequencing. Image created with BioRender.com. Publication and licensing rights provided through agreement number VG256RG3L2. **b** Number of mutations per isolate of WT or *rad30*Δ yeast accrued following no UV (*n* = 1 and 2 for WT and *rad30*Δ), 9×(*n* = 25 and 15 for WT and *rad30*Δ), or 15×(*n* = 46 and 50 for WT and *rad30*Δ) exposures to UVC light (25 J/m^2^). Mutations were identified by genome sequencing of each independent yeast isolate and indicated. The median number of UV-induced mutations from WT and *rad30*Δ yeast following 15× exposures of UVC was compared by two-sided Mann–Whitney rank sum test. ****p < 0.0001.

To examine the involvement of pol η in the formation of atypical UV-induced mutations, we generated homozygous diploid yeast strains lacking pol η (*rad30*Δ*/rad30*Δ) and subjected them and corresponding WT diploid yeast to either 9 or 15 serial exposures of approximately 25 J/m^2^ UVC (Fig. 1a) to accumulate UV-induced mutations as previously reported^8^. Following UV exposure, 65 independent clones were isolated and subjected to whole genome sequencing to identify single and tandem base pair substitutions. Compared to WT diploid yeast, pol η-deficient yeast exposed to 15 rounds of UVC light had a 1.26-fold elevated median substitution density (calculated by dividing the median number of UV-induced substitutions in *rad30*Δ*/rad30*Δ yeast genomes by the corresponding median number in WT genomes) indicating that in general, pol η protects slightly against UV-induced mutations (Fig. 1b).

**Fig. 2:**
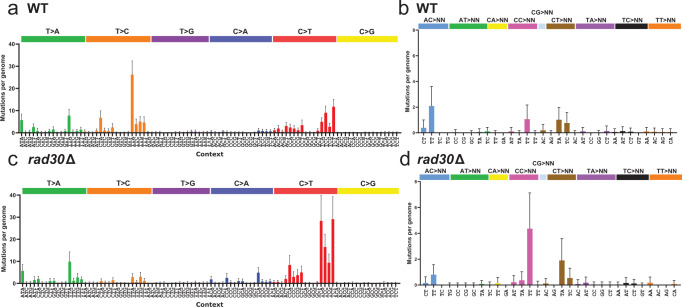
Mutation spectra from whole genome sequencing of UVC irradiated WT and *rad30*Δ yeast. **a** Mutation profile of single-nucleotide substitutions in UVC-exposed (25 J/m^2^) WT yeast (exposed 15x). Data are presented as mean values ± SD of the mutation count for each substitution type (e.g., C > A, C > G, etc.) and trinucleotide context is depicted. The middle base of each trinucleotide context is mutated. Sequencing and mutation spectra for these samples was previously published^8^. **b** Mutation profile of tandem nucleotide substitutions in UVC-exposed WT yeast. Data are presented as mean values ± SD. **c** Mutation profile of single-nucleotide substitutions in UVC-exposed *rad30*Δ yeast. Data are presented as mean values ± SD of the mutation count per genome for each substitution type (e.g., C > A, C > G, etc.) and trinucleotide context is depicted. The middle base of each trinucleotide context is mutated. **d** Mutation profile of tandem nucleotide substitutions in UVC-exposed *rad30*Δ yeast. Data are presented as mean values ± SD. The UV-induced mutation spectra of WT (*n* = 45) and *rad30*Δ (*n* = 49) yeast was compared by two-sided *χ*^2^ analysis. Spectra show mutations from 45 *(*WT) and 49 (*rad30*Δ) independent biological isolates. Individual data points not shown to maximize clarity.

Evaluation of the UV-induced mutation spectra, however, revealed significant differences upon pol η loss (*p* < 0.0001 by two-sided *χ*^2^ analysis comparing mutation spectra of Fig. 2a, c). As previously reported^8^, WT yeast displays canonical UV signature mutations (i.e. TC > TT, CT > TT, CC > CT, CC > TC, and CC > TT) as well as non-canonical T > C, T > A, and AC > TT UV-induced mutations (Fig. 2a, b). *rad30*Δ*/rad30*Δ yeast displayed a higher rate of the canonical UV mutations (Fig. 2c), which is expected as pol η is reported to be error-free in synthesizing past CPDs in TT dipyrimidines^18^. It is likely that pol η drives similar non-mutagenic synthesis across CPDs in other dipyrimidines and suggests that most mutations caused by CPDs result from elevated cytidine deamination within the lesion^22^. Loss of pol η nearly abolished T > C substitutions (Fig. 2c), indicating that they are induced solely by a lesion that the TLS polymerase mutagenically bypasses. Yeast pol η has been previously shown to cause T > C substitutions at site-specific 6-4 PPs^16^. These results indicate that 6-4 PPs likely cause the T > C mutations in UV-irradiated WT yeast. *rad30*Δ*/rad30*Δ yeast also displayed respective 3- and 2-fold elevation of CC > TT and CT > TA tandem substitutions, consistent with their induction by CPDs. In contrast, a 2-fold reduction in the number of atypical AC > TT mutations per genome was observed (Fig. 2d), indicating that like for 6-4 PPs, pol η is mutagenic in the bypass of AC lesions. Pol η-deficiency had no effect on T > A mutations, indicating that other polymerases are responsible for T > A substitutions in yeast. Notably, C > A substitutions in CA dinucleotide contexts also were elevated in *rad30*Δ*/rad30*Δ yeast, highlighting that pol η is protective against an additional UV-induced lesion (Fig. 2c). Pol η may additionally function in bypass of other potentially mutagenic UV-induced DNA lesions that could not be observed in our whole genome sequencing dataset. We assessed NER-proficient yeast because yeast deficient in both NER and pol η are extremely sensitive to UVC exposure (which precludes repeated UVC exposure and mutation accumulation) and to more closely model the human XPV condition (Supplementary Fig. 1). Therefore, UV lesions that are rapidly repaired by NER and rarely encounter TLS polymerases will be under-represented in our analysis. Nonetheless, the UV-induced mutation spectrum of *rad30*Δ*/rad30*Δ yeast was still significantly different than for yeast deficient in GG-NER (i.e. *rad16*Δ*/rad16*Δ^8^) or TC-NER (i.e. *rad26*Δ*/rad26*Δ; Supplementary Fig. 2a, b) (*p* < 0.0001 by two-sided *χ*^2^ analysis comparing mutation spectra of Fig. 2c and Supplementary Fig. 2a), which displayed similar distributions of UV-induced substitution types in sequence contexts as those observed in WT yeast.

### UVC and UVB exposure produce similar mutation spectra in *rad30*Δ*/rad30*Δ yeast

**Fig. 3:**
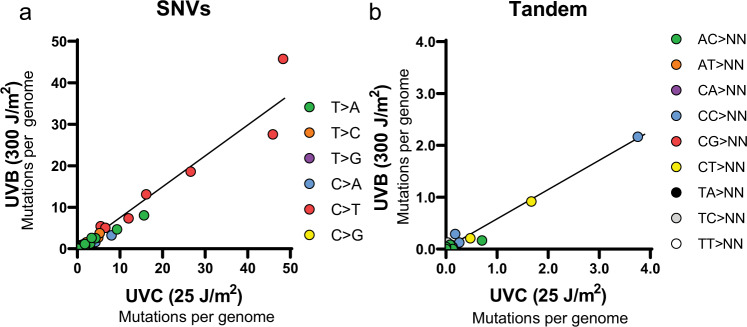
UVC induced mutations correlates with UVB induced mutations. **a** Single mutation counts in yeast irradiated with 25 J/m^2^ UVC compared to yeast irradiated with 300 J/m^2^ UVB. Pearson’s coefficient shows strong correlation. Rho = 0.97, *p* < 0.0001. **b** Tandem mutation counts in yeast irradiated with 25 J/m^2^ UVC compared to yeast irradiated with 300 J/m^2^ UVB. Correlation of mutation types caused by the two UV wavelengths was assessed by Pearson’s coefficient (Rho = 0.81, *p* < 0.0001).

UV light is composed of three subtypes: UVA, UVB, and UVC. While UVC light has traditionally been used in experiments designed to evaluate the formation and repair of CPDs and 6-4 PPs due to its more efficient production of these lesions, almost all solar UVC light is absorbed by the atmosphere, limiting its relevance for damaging cellular DNA. Instead, most organisms are exposed to UVA and UVB light, the latter of which is likely most responsible for causing UV-induced photoproducts in cells exposed to solar irradiation^23–25^. To confirm that the mutations resulting from exposure to UVC light are reflective of the more physiologically relevant UVB exposure, we repeated 15 serial exposures of *rad30*Δ*/rad30*Δ yeast to 300 J/m^2^ of UVB light and subsequently sequenced the genomes of 23 independent clonal isolates. Exposure to the higher UVB dose yielded a similar number of mutations per genome compared to the 25 J/m^2^ UVC exposure (Supplementary Fig. 3). Moreover, full single and tandem mutation spectra from UVB exposed *rad30*Δ*/rad30*Δ yeast bore striking resemblances to those obtained after UVC-exposure (Fig. 3). Direct comparison of either the abundance of single substitutions per genome occurring in each possible trinucleotide context or the number of each type of tandem substitution observed per genome caused by UVB and UVC light showed a very high concordance (Pearson Rho = 0.97; *P*-value < 0.0001), indicating that the wavelength of UV light did not significantly alter the types of mutations observed, just the efficiency at which overall lesions were formed.

### Transcriptional and replicative asymmetry of mutation spectra in UV-irradiated yeast

**Fig. 4:**
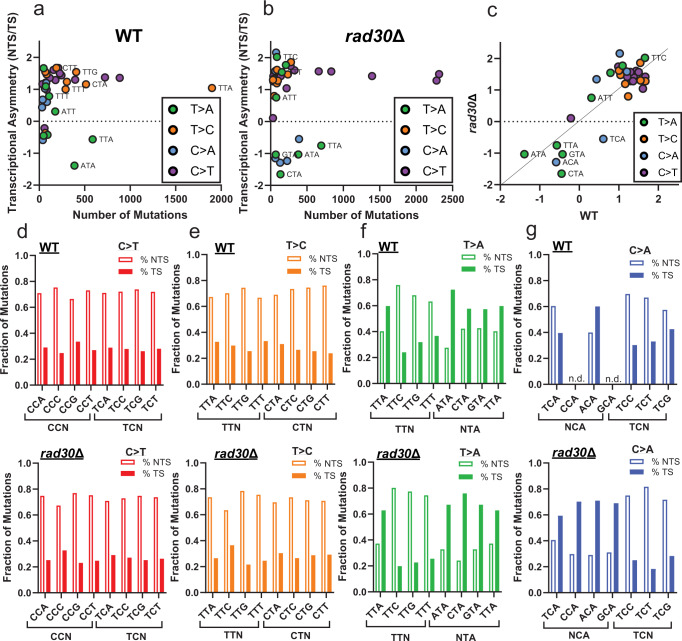
Transcriptional strand bias of UV-induced mutations in WT and *rad30*Δ yeast. **a** Transcriptional asymmetry (i.e., normalized ratio of mutations on NTS relative to TS across all yeast genes) is plotted relative to total number of mutations for each trinucleotide context in WT yeast. Only mutation classes with at least 30 mutations are plotted. The color of the circle indicates the mutation class (e.g., C > T). Statistical significance of transcriptional asymmetry in each mutation class and sequence context with at least 30 mutations was determined by a two-sided *χ*^2^ analysis using the expected number of mutations to be associated with the NTS and TS strands (determined by calculating the frequency of trinucleotide contexts on each strand). The resulting *p*-values were corrected for multiple hypothesis testing using the Bonferroni method and listed in Supplementary Data 4. **b** Same as A, except for *rad30*Δ yeast. **c** Strand bias of UV-induced mutations in WT yeast versus the bias in *rad30*Δ yeast. Fraction of (**d**) C > T, (**e**) T > C, (**f**) T > A, and (**g**) C > A mutations occurring on the non-transcribed strand relative to the transcribed strand for the indicated trinucleotide contexts in WT (top) and *rad30*Δ yeast (bottom). N.d. = not determined. Bars for transcriptional asymmetry are representative of the percentage of total mutations occurring on either the NTS or TS for a given substitution pair. As such, there is no variation.

UV-induced mutations that result from bulky UV photoproducts exhibit transcriptional asymmetry due to TC-NER preferentially repairing lesions on the transcribed strand (TS) of genes prior to replication^26^. The role of TC-NER in generating this asymmetry can be confirmed as yeast deficient in TC-NER (i.e. *rad26*Δ*/rad26*Δ yeast) have low transcriptional strand bias^8^ (Supplementary Fig. 2c–g). Observed transcriptional asymmetry of UV-induced mutations provides support that lesions are likely UV photoproducts and indicates on which of the complementary dinucleotide bases the lesion was formed. We therefore assessed transcriptional asymmetry of mutations in pol η-deficient yeast to evaluate whether loss of this polymerase influenced the strand association of UV-induced mutations. The expected favoring of the non-transcribed strand (NTS) for canonical C > T mutations was apparent in *rad30*Δ*/rad30*Δ yeast (Fig. 4d), emphasizing the impact of TC-NER. Similar to those in WT yeast^8^, the limited number of T > C substitutions generated in *rad30*Δ*/rad30*Δ yeast also occurred preferentially on the NTS (Fig. 4e), confirming that these substitutions still likely result from UV-induced 6-4 PPs that are mutagenically bypassed by another TLS polymerase, but at a lower efficiency. In both WT and *rad30*Δ*/rad30*Δ yeast, T > A substitutions in an NTA context occurred more abundantly on the TS, indicating that these are primarily A > T substitutions at these contexts (Fig. 4f). C > A substitutions in the NCA context, which were formed exclusively in *rad30*Δ*/rad30*Δ yeast, also displayed bias for the TS (Fig. 4g). This transcriptional asymmetry indicates that like for T > A substitutions, C > A mutations originate from lesions on the opposite DNA strand. Thus, C > A mutations are in fact G > T substitutions occurring in TGN contexts and are either indicative of a novel TG photoproduct inducing TC-NER, or are another UV-induced non-photoproduct lesion which is only observed in the absence of Pol η. Supporting the latter hypothesis, UV light is known to induce oxidative damage, including 8-oxoguanine (8-oxoG)^27^, which results in G > T substitutions and can favor TGN sequence contexts^28,29^. Additionally, pol η is known to play an important role in the non-mutagenic bypass of 8-oxoG lesions in yeast^30,31^.

A recent study identified replicative asymmetry (i.e. mutations occurring primarily on the leading strand or the lagging strand during replication) among melanoma mutations that are mostly UV-induced^32^. C > T substitutions among this dataset showed a lagging strand template bias, which was attributed to more mutagenic TLS occurring during lagging strand synthesis. We reasoned that our mutation accumulation experiments in WT and *rad30*Δ*/rad30*Δ yeast would provide an experimental test of whether TLS preferentially occurs on the lagging strand. Using a previously established method for assessing replicative asymmetry of mutations in yeast whole genome sequencing^33,34^, we profiled the relative ratio of complementary UV-induced substitutions as a function of the distance of the mutation’s position to the closest origin of replication. From these plots, preferential mutagenic TLS associated with either the leading or lagging strand is evident as a greater abundance of a specific substitution than its corresponding complementary mutation type in regions with leftward moving forks (i.e. downstream of origins), while the inverted relationship occurs in regions with rightward moving forks (i.e. upstream of origins). This relationship results in a characteristic “cross” in the graph of the fractional abundance of complementary mutation types, with the slope of each line indicating how much a given strand is favored. In WT yeast, T > A and T > C mutations displayed no detectible replicative asymmetry, while C > T substitutions occurred with slight preference to the leading strand (Supplementary Fig. 4). These patterns suggest that TLS in yeast occurs nearly evenly during synthesis of the leading and lagging strands. A similar lack of replication asymmetry was observed in pol η-deficient yeast. This result indicates that even in the absence of pol η, pol ζ-mediated TLS occurs equally on both DNA strands.

### Translesion synthesis polymerases η and ζ coordinate to bypass atypical UV lesions in yeast

To investigate the involvement of other polymerases in the formation of the atypical mutations, we created novel single-stranded reversion reporters that contain reversion sequences matching the atypical substitution patterns. The reporters were designed proximal to the ChrV telomere in yeast strains (ySR585, ySR586) that contain the temperature sensitive *cdc13-1* allele, which at non-permissive temperature (37 °C) results in telomere un-capping, 5’ to 3’ end resection, and single-stranded DNA formation near telomere ends^35^.

**Fig. 5:**
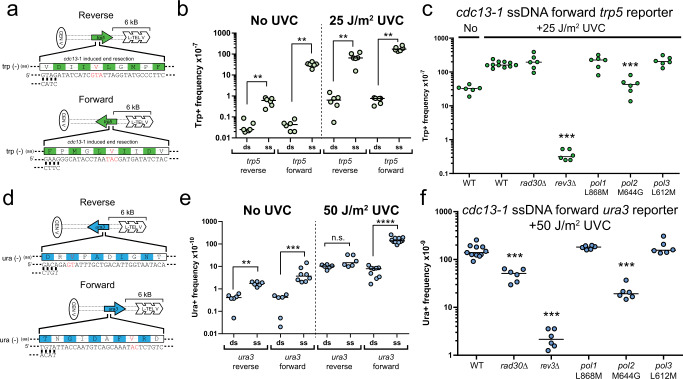
Specific DNA polymerases alter the mutation frequency of the A > T and AC > TT substitution in single stranded reporter system. **a** Single-stranded reporter schematic of the *trp5* reporter with the E50V mutation. Reverting bases are shown in red. **b** Six independent biological measurements of the frequency of Trp+ colonies after incubation without UV exposure or exposed to 25 J/m^2^ of UVC. Induction of mutations occurs in the *cdc13-1* ssDNA strains (ss) containing either orientation of the *trp5* reporter. **p = 0.0022 (**c**) Frequency of the T > A substitution is significantly reduced in *rev3*Δ and *pol2* M644G yeast. Median Trp+ reversion frequencies were determined from six biologically independent measurements for yeast treated with 25 J/m^2^ UVC light. **d** Schematic of the *ura3* reversion reporter with the K93V mutation. Reverting bases and codons are shown in red. **e** Six biologically independent measurements of the frequency of Ura+ colonies after incubation with and without exposure to 50 J/m^2^ of UVC for *CDC13* (ds) and *CDC13*−1 (ss) strains. **p = 0.0022, ***p = 0.0007, ****p < 0.0001, n.s.= not significant. **f** Frequency of the AC > TT substitution is significantly reduced in *rev3*Δ*, rad30*Δ, and *pol2* M644G yeast. Median Ura+ reversion frequencies were determined from six biologically independent measurements for yeast treated with 50 J/m^2^ UVC light. Statistical significance was determined in (**c**) and (**f**) using pairwise two-tailed Mann–Whitney ranked sum tests to independently compare the median reversion frequency of each UVC-treated yeast stain harboring polymerase deficiency to the UVC-treated WT strain. Open circles represent each independent sample. ***p = 0.0002.

To test polymerases involved in mutagenic bypass of the TA photoproduct that results in A to T substitutions, we created an inactive variant of the *TRP5* gene which carried a *trp5* E50V mutation^36^ such that the coding and non-coding strands of the gene contained GTA and TAC sequences, respectively at the reversion site. Insertion of this *trp5* E50V near the telomere end in ChrV in *cdc13*-*1* yeast, allows one strand of the reporter to be resected and leaves the remaining strand unambiguously the target for UV-induced damage that results in reversion. By inserting *trp5* E50V in both orientations, either the GTA or TAC lesion context can be evaluated. The *trp5* E50V reversion reporter could theoretically be reverted by either a T > A or TA > AG substitution in the GTA sequence orientation or A > T and TA > CT substitutions in the TAC orientation (i.e. GTA to GAA or GAG codon changes) to produce the required valine to glutamic acid change (Fig. 5a). UV-light-induced *TRP5* reversion with the reporter in either orientation over non-treated yeast; however, reversion rates were 100-fold higher in the *cdc13*-*1* strains, likely due to impaired lesion repair or increased lesion formation in ssDNA (Fig. 5b). Sequencing of UV-treated revertants revealed that regardless of orientation, only single base substitutions occurred, indicating that UV light can induce a T > A substitution (potentially at a GT or TA photoproduct) nearly as efficiently as an A > T substitution at the TA photoproduct. We subsequently generated *rad30*Δ and *rev3*Δ mutations in the *cdc13*-*1* forward orientation strains to assess the impact of TLS polymerase deficiency on A > T substitutions generated during the bypass of a TA photoproduct. In the forward orientation (A > T), *rad30*Δ resulted in no detectible change in reversion frequency, consistent with the similar abundance of T > A mutations in TA contexts observed in UV-irradiated WT and *rad30*Δ whole genome sequenced yeast (Fig. 5c). In contrast, *rev3*Δ reduced reversion >100-fold, even 44-fold lower than the untreated *cdc13*-*1* strain. This drastic reduction in mutation rate indicates that pol ζ is absolutely required for A > T substitutions at TA photoproducts, potentially by both error-prone insertion across from the lesion as well as extension from the nucleotide across from the damage.

We next assessed the requirements for pol η and pol ζ in UV-induced AC > TT substitutions. Through a similar experimental strategy, we inserted our previously described *ura3* K93V allele that allows for reversion to *URA3* through an AC > TT tandem substitution^8^ into the subtelomere of ChrV in both orientations of *CDC13* + and *cdc13*-*1* temperature sensitive yeast (Fig. 5d). Because AC > TT mutations occur less frequently than the A > T substitutions measured with the *trp5* reporter, strains containing the *ura3* reporter were exposed to 50 J/m^2^ UVC. No revertants were observed in the absence of UVC regardless of the reporter orientation or whether the reporter was single-stranded or double stranded DNA. For each of these “no UVC” controls, we therefore calculated a maximum estimated reversion frequency as the frequency for a single revertant to have been observed among the number of yeast cells plated in each experiment. With UVC-exposure, the single-stranded reporter in the forward orientation (containing an AC dinucleotide at the reversion site) showed a 30-fold elevation in reversion frequency compared to the estimated maximum frequency for non-UVC exposed yeast temperature shifted to 37 °C (Fig. 5e). In contrast, UVC irradiation only elevated the reversion frequency of the reverse orientation reporter with the AC dinucleotide in the resected strand 8-fold. The increase in the reverse orientation is likely due to incomplete resection of our reporter among cells in the culture as a similar Ura+ frequency was observed with UVC-irradiated double stranded reporters. Similarly to the *trp5* reporter, the *ura3* reporter could revert by two possible codon changes to create a functional *URA3* allele (i.e. GTA to AAA or AAG), allowing the potential detection of GT > AA or GTA > AAG substitutions in the reverse orientation and AC > TT or TAC > CTT substitutions in the forward orientation. Sequencing of *URA3* revertants from the reporter in either orientation revealed only tandem AC > TT mutations. These results confirm that the primary lesion producing AC > TT tandem substitutions is an atypical AC photoproduct as these substitutions occur much more frequently when the AC containing strand in the reporter is present. The frequency of the AC > TT reversion was also measured in various polymerase mutant or knockout lines to identify contributing polymerases (Fig. 5f). The absence of pol η resulted in a 3-fold decrease in the mutation frequency of AC > TT in the single-stranded reporter, consistent with the whole-genome sequencing evidence that pol η contributes to mutations at AC photoproducts (Fig. 2c). Significantly, the *rev3*Δ strain had at least a 65-fold reduction (no Ura+ colonies were observed for *rev3*Δ strain; fold change was determined using the maximum estimated reversion frequency) compared to the UV-treated single-stranded control strain with functional TLS. Moreover, the reversion rate in UV treated *rev3*Δ yeast was indistinguishable from untreated WT yeast with the reporter containing the target AC in ssDNA, indicating that as with A > T bypass of the TA photoproduct, AC > TT tandem substitutions were completely dependent on pol ζ. The partial reduction of reversion in pol η-deficient yeast and complete loss of reversion upon pol ζ-deficiency indicates that AC > TT mutagenesis frequently proceeds in a two-polymerase mechanism where pol η mutagenically inserts at least one of the nucleotides at the AC photoproduct, while pol ζ extends from the pol η insertion to complete TLS.

The persistence of some reversion events for both the A > T and AC > TT reporters in the absence of pol η, indicates that either pol ζ is capable of completing both the insertion and extension steps of mutagenic bypass of the TA and AC photoproducts, or another DNA polymerase is involved in the insertion at the lesion site. We reasoned that a replicative polymerase would be responsible for such an activity as the only other TLS polymerase in yeast is Rev1, which exclusively incorporates deoxycytidine triphosphates and therefore would not be a candidate for either substitution type. To gain insight into whether any of the replicative polymerases impact UV-induced A > T and AC > TT substitutions, we generated the nucleotide selectivity mutants of *POL1* (L868M), *POL2* (M644G), and *POL3* (L612M)^19–21,37^ in the strains containing our single stranded reversion reporters and assessed the ability of these change of spectrum mutators to alter reversion rates. Creation of these alleles imparted a clear spontaneous mutator phenotype to the yeast as observed by increased canavanine resistance indicating elevated mutations in the *CAN1* gene (Supplementary Fig. 5). However, neither the *pol1* L868M mutant nor the *pol3* L612M mutant altered UV-induced reversion of either the *trp5* A > T reporter or the *ura3* AC > TT reporter (Fig. 5c, f). Surprisingly, the *pol2* M644G strain decreased both the A > T and AC > TT reversion frequency 5- and 10-fold respectively compared to UV-irradiated ssDNA controls. These results highlight a potential role for DNA pol ε in inserting across from UV damage where the *pol2* M644G allele allows pol ε to insert non-adenine bases more efficiently across from TA or AC photoproducts, resulting in a reduction of revertants.

## Discussion

Our cumulative data highlight distinct roles of replicative and TLS DNA polymerases in UV-induced mutagenesis in yeast. Whole genome sequencing of UV-exposed *rad30*Δ/*rad30*Δ yeast indicates that pol η accurately bypasses CPD lesions that are thought to cause C > T substitutions in dipyrimidines, while mutagenically bypassing 6-4 PPs to cause T > C substitutions, consistent with previous reports^18,38^ (Fig. 2). Pol η also appears to have low fidelity when bypassing the putative lesion responsible for AC > TT mutations, resulting in fewer UVC-induced AC > TT substitutions in *rad30*Δ/*rad30*Δ yeast than in wild-type yeast. The reduction in both substitution types in *rad30*Δ/*rad30*Δ yeast indicates that either an accurate pol η-independent bypass exists for the responsible lesions, or that slowed bypass in the absence of pol η may allow greater repair of the lesions by NER. Unlike for the 6-4 PP-associated T > C substitutions, however, the AC > TT substitution is not completely dependent on pol η, indicating other DNA polymerases likely participate in mutagenic bypass. While 6-4 PPs require both pol η and pol ζ to be present to mutagenically bypass the lesion^18,39^, AC lesions appear to have greater flexibility in polymerase choice. The results from our reversion assays indicate that pol ζ alone or in concert with pol η and/or mutated pol ε can all mediate bypass. The absence of pol η had no effect on the T > A substitution (in either whole genome sequencing or reversion assays), indicating that in yeast pol η is not involved in TA photoproduct bypass. Instead, T > A reversion only decreased in strains with mutated pol ε or pol ζ-deficiency, suggesting TA photoproducts could be bypassed by the coordinated activity of pol ε or pol ζ, or in some instances by pol ζ alone. Previous in vitro studies have provided evidence that both the replicative polymerases δ and ε maintain the biochemical capacity to participate in translesion synthesis past weakly blocking template strand damage^40,41^. Our results suggest that pol ε, but not pol δ, contributes to bypass of UV-induced mutations in vivo. However, we cannot exclude the possibility that the pol δ steric gate mutation (*pol3* L612M) failed to significantly change either the nucleotide selectivity or the frequency of insertion across from AC or TA photoproducts, which would be needed for detection in this assay. The *pol2* M664G allele also reportedly increases dNTP pools in yeast, which could indirectly alter the bypass fidelity of pol η or pol δ and contribute to the observed reduction of reversion frequency. We expect that a similar indirect mechanism of altered lesion bypass would be absent in human cells as they do not experience increased dNTP levels upon DNA damage^42–44^. Finally, the evaluation of mutation induction during telomere re-synthesis in our yeast system as opposed to during replication could also impact polymerase usage during lesion bypass.

Interestingly, UV-irradiated *rad30*Δ/*rad30*Δ yeast also had an increase in C > A substitutions occurring at CA dinucleotides (Fig. 2) compared to wild-type yeast. The transcriptional asymmetry of these mutations reveals that they are likely G > T substitutions occurring at TG sequences on the non-transcribed strand (Fig. 4g). The sequence specificity of these substitutions may indicate that they are formed by a novel UV photoproduct occurring at TG sequences that pol η can bypass in an error-free manner. Alternatively, pol η has been previously shown to accurately bypass 8-oxoG lesions caused by oxidative damage^45,46^. Some evidence suggests 8-oxoG may be repaired by TC-NER^47^, while its mutagenic bypass results in G > T substitutions consistent with the observed lesions in UV-treated *rad30*Δ/*rad30*Δ yeast. However, the known sequence preferences for guanine oxidation^48^ is unlikely to be specific enough to account for the UV-induced G > T substitutions in TG dinucleotides observed in *rad30*Δ yeast. Therefore, for 8-oxoG lesions to cause these mutations, their sequence specificity would need to be derived from mutagenic TLS bypass instead of lesion formation. One potential mechanism may be that, in the absence of pol η, an alternative polymerase induces mutation through a primer-template slippage event in which the 5’T neighboring the 8-oxoG is used to direct insertion of an adenine followed by re-alignment of the strands and continued synthesis^49^. However, mutations in *ogg1*Δ *rad30*Δ yeast, which would result from error-prone bypass of 8-oxoG, lack the same sequence specificity^50^ as the UV-induced G > T mutations in our *rad30*Δ dataset, leaving the origin of these mutations in question.

Loss of pol η in human cells results in XPV^16^, a disease that increases the risk of skin cancer by 10,000-fold^14^. The mutation load in XPV cells is significantly higher than normal^51^, which is expected to be a large contributor to the higher incidence of tumor development. Mutation spectra from the *HPRT* gene in human cells lacking pol η significantly differed from the spectra of wild-type cells, in that the former have higher incidence of transversion substitutions^52^. While only a limited number of *HPRT* mutants were sequenced (45 total for XPV irradiated in G1 and S phase combined), 16 involved G > T substitutions, the majority (11) of which occurred at TG dinucleotides^52^. These results suggest that the elevation of G > T substitutions in UVB and UVC-irradiated *rad30*Δ/*rad30*Δ yeast is conserved in irradiated XPV human cells and may even be more prominent. Whether the other aspects of the *rad30*Δ yeast UV-spectrum are conserved remains unknown. Significant differences may exist as human cells have a larger number of TLS polymerases that could participate in UV lesion bypass in the absence of pol η^17,53^. Human pol ι has been implicated in the altered, and more severe, mutation spectra found in XPV cells^54^. Likewise, pol κ and pol θ are implicated in TLS bypass of UV-induced mutations and could establish other human-specific aspects of the UV-induced mutation spectrum^55^. Interestingly, recent whole genome sequencing of UVA-treated XPV cells primarily revealed mostly C > T and some C > A substitutions in dipyrimidine sequences, suggestive of CPD-induced mutagenesis^50,56,57^. The drastic difference in mutation spectrum obtained from UVA-treated XPV cells compared to mutations in the *HPRT* spectrum of UVC-treated XPV cells suggests that in pol η-deficient human cells, the lower energy UVA light likely induces significantly different mutagenic lesions than UVC.

Understanding which aspects of the mutation spectrum in XPV individuals that give rise to their elevated rate of skin cancer will require concerted effort, including the sequencing of multiple skin cancers from XPV individuals to determine the specific driver mutations acquired. The data provided from UV-irradiated *rad30*Δ yeast suggest that driver mutations in XPV tumors likely involve canonical UV-signature mutations (i.e. C > T substitutions in dipyrimidines and CC > TT tandem substitutions) as opposed to non-canonical mutations (i.e. AC > TT or TA > TT substitutions). Additionally, C > A substitutions may play a more significant role in driving carcinogenesis within these tumors. While the incidence of all skin cancers is elevated in XP individuals, basal cell carcinomas generally occur earlier than melanomas. Such a difference in age of onset could partially be explained by the most common activating mutations in these two cancer types, *SMO* W535L (a C > A substitution)^58^ and *BRAF* V600E/K (A > T or AC > TT substitutions) involving UV lesions disparately impacted by loss of pol η.

## Methods

### Yeast strains

All strains listed in Supplementary Data 1. The *rad30*Δ/*rad30*Δ strain (yML256) was created in the BY474 yeast strain by replacing one *rad30* allele with *KANMX*, and the other *rad30* allele with the *LEU2* marker. Reversion strains were constructed in the yeast strain ySR127^59^, which contains *ADE2*, *URA3*, and *CAN1* deleted from their normal chromosomal positions and re-inserted as an array into the *LYS2* gene positioned near the left telomere of chromosome 5. The *ura3* AC > TT reversion mutation was generated by amplifying the mutant gene from yDM04^8^ (MATa/α *his7*-*2*/*his7-2 ura3Δ/ura3Δ can1Δ/can1Δ ade2Δ/ade2Δ leu2-3,112/leu2-3,112 trp1-289/trp1-289 lys2::ADE2-URA3-CAN1/ lys2::ADE2- ura3-K93V-CAN1*) with primers that contain flanking regions of homology to the triple reporter in either the forward or reverse orientation. These fragments were then transformed into yeast, which were then screened for *ura3*-deficiency on SC media containing 5-fluoroorotic acid (5-FOA). Proper editing of *URA3* within these isolates was confirmed by isolating total genomic DNA from the strains, PCR amplifying the *ura3* gene using the KOD hotstart kit from Sigma Aldrich (Cat #71086), and Sanger sequencing the resulting PCR product. *Trp5 CDC13* GAA > GTA reversion strains were generated as previously described (yHSM1)^8^. The *cdc13*−1 strains were generated by mating YML386.24 with yBV43/yBV44 and selecting spores that contained the *cdc13*−1 allele and the mutation in *trp5*. Polymerase mutants were generated by either a deletion cassette or CRISPR/Cas9 targeted mutagenesis. Guide RNAs were designed using a database for yeast gRNAs: wyrickbioinfo2.smb.wsu.edu/crispr.html and oligos used for the repair templates contained the desired mutation along with a mutated PAM site (All oligonucleotides listed in Supplementary Data 2).

### Whole genome sequencing of irradiated yeast

Diploid yeast cultures were grown to late log phase in YPD medium. Cells were collected via centrifugation and resuspended in sterile water to an approximate cell density of 1 × 10^7^ cells/mL. 3-5 μL aliquots of resuspended yeast were independently spotted into arrays on YPD plates. Plates were exposed to 25 J/m^2^ of UVC radiation, or 300 J/m^2^ of UVB, and then incubated in the dark for 1 to 2 days at 30 °C. Cells from each individual spot were then re-suspended in sterile water to ~1 × 10^7^ cells/mL or less, re-spotted to fresh YPD plates, and irradiated again at the same dose. Following fifteen rounds of UV passaging, yeast patches were struck for isolation onto YPD plates and incubated at 30 °C until growth appeared. Overnight cultures were started either directly from isolates, or from isolates that were patched to fresh YPD plates, and grown overnight in YPD media. Cells were then harvested by centrifugation, resuspended in DNA lysis buffer (2% Triton X-100, 1% SDS, 100 mM NaCl, 1 mM EDTA, 10 mM Tris-HCl pH 7.5–8.0) and vortexed in Tris buffered Phenol:Chloroform:Isoamyl alcohol (PCI, Fisher) with acid washed 425-600 mm glass beads (Sigma). Following bead beating, 1 × TE buffer (10 mM Tris 1 mM EDTA, pH 7.5–8.0) was added to the samples and they were centrifuged to induce separation of the aqueous phase which was then removed and precipitated with 100% ethanol. Pellets were washed with 70–80% ethanol and resuspended in 0.1–1 × TE buffer. Samples were then incubated with RNAse A at 37 °C to remove contaminating RNA and subjected to another round of PCI extraction^8^. An Illumina sequencing library was prepared from each genomic DNA using a Kapa Hyper preparation kit (Roche; cat# KR0961) and multiplexed libraries sequenced on an Illumina Hiseq 4000 platform.

### Analysis of yeast mutation data

Fastq reads from the clones were aligned to the Saccer3 reference genome and mutations (Supplementary Data 3) were called by allelic fraction using the CLC Genomics Workbench version 7.5 (Qiagen) similarly to previous reports^8^. The trinucleotide context of the substitutions was pulled from the reference genome using BEDtools^60^, and the single and tandem substitutions were subsequently quantified within each unique trinucleotide context. Mutations were separated by single or tandem substitutions and complimentary contexts were combined using custom Python 3 and RStudio scripts. Mitochondrial mutations were excluded. Transcriptional asymmetry was assessed using BEDtools intersect function to cross reference mutation coordinates with published transcription start sites (TSS) and transcription end sites (TES) in yeast found at https://hgdownload.soe.ucsc.edu/goldenPath/sacCer3/bigZips/genes. Mutations on each strand were quantified using a custom RStudio script. Replicative asymmetry was assessed using established coordinates of genes and origins of replication^61^. Mutations were placed into groups based upon whether they occurred in genes transcribed on the top or bottom DNA strand and their location in relationship to the percent distance between neighboring origins. Statistical analyses were performed in RStudio, Python3, or GraphPad Prism6.

### *URA3* reversion reporter assay

To calculate the frequency of the UV induced K93V mutation in the different yeast strains, cells were grown to saturation and then diluted 1:10 in YPDA. The diluted cells were then temperature shifted and grown at 37 °C for 6 h to allow for telomere resection. Cells were then collected by centrifugation, resuspended in sterile water, plated onto SC-URA media, and exposed to 50 J/m^2^ UV light in a dark room. Plates were incubated at 23 °C for 7–10 days. Serial dilutions were also made of the cell suspension and plated on SC plates to calculate the actual number of surviving cells plated. Rate of reversion was calculated by dividing the number of revertants by the total number of surviving cells. Statistical comparisons of reversion rates were assessed using GraphPad Prism 6. Revertant colonies were patched onto YPDA for subsequent genomic DNA isolation. Cells were resuspended in lithium acetate lysis buffer (20 mM Tris-HCl, 200 mM LiAc, 1.5 % SDS, pH 7.4) with glass beads (Sigma cat# G8772), vortexed, and incubated at 65 °C. Samples were vortexed and 4 M NaCl was added prior to centrifugation to separate the precipitate. The supernatant was transferred to a new tube and binding buffer (25 mM tris-HCl pH 6.0, 5.5 M GuNaSCN) was added prior to column purification (Omega Bio-tek; cat # DNACOL-02). The column was washed with wash buffer (10 mM tris-HCl, pH 8.0, 80% ethanol) and eluted with 1× TE buffer (10 mM Tris-HCl, pH 8.0). Purified genomic DNA was amplified using the KOD hotstart DNA polymerase kit (Sigma Aldrich; cat # 71086) and sequenced to verify the reversion (see Supplementary Data 2 for oligonucleotides).

### *TRP5* reversion reporter assay

To calculate the frequency of the UV induced T > A mutation in the different yeast strains, cells were grown to saturation and then diluted 1:10 in YPDA. The diluted cells were then temperature shifted and grown at 37 °C for 6 h to allow for telomere resection. Cells were then collected by centrifugation, resuspended in sterile water, plated onto SC-TRP media, and exposed to 25 J/m^2^ UV light in a dark room. Plates were incubated at 23 °C for 5 days. Serial dilutions were also made of the cell suspension and plated on SC plates to calculate the actual number of surviving cells plated. Rate of reversion was calculated by dividing the number of revertants by the total number of surviving cells. Statistical comparisons of reversion rates were assessed using GraphPad Prism 6. Revertant colonies were patched onto YPDA for subsequent genomic DNA isolation. Cells were resuspended in lithium acetate lysis buffer (20 mM Tris-HCl, 200 mM LiAc, 1.5 % SDS, pH 7.4) with glass beads (Sigma cat# G8772), vortexed, and incubated at 65 °C. Samples were vortexed and 4 M NaCl was added prior to centrifugation to separate the precipitate. The supernatant was transferred to a new tube and binding buffer (25 mM tris-HCl pH 6.0, 5.5 M GuNaSCN) was added prior to column purification (Omega Bio-tek; cat # DNACOL-02). The column was washed with wash buffer (10 mM tris-HCl, pH 8.0, 80% ethanol) and eluted with 1x TE buffer (10 mM Tris-HCl, pH 8.0). Purified genomic DNA was amplified using the KOD hotstart DNA polymerase kit (Sigma Aldrich; cat # 71086) and sequenced to verify the reversion (see Supplementary Data 2 for oligonucleotides).

### CAN1 mutation assay

Mutation frequencies were determined for each replicative polymerase mutant. Yeast were plated to ~200 cells per plate on YPDA media and grown at 23 °C for 3 days. Independent colonies were then resuspended in water, serially diluted, and plated on SC media or SC media supplemented with 0.006% canavanine and grown for 3 days at 23 °C. The frequency of Can^R^ mutants was calculated by multiplying the number of Can^R^ colonies by the dilution plated on SC media, divided by the number of colonies on the SC plate multiplied by the dilution plated on the canavanine containing plates.

### UV survival assay

Yeast strains were grown overnight in YPDA medium, spun down, and resuspended in water. Serial 1:10 dilutions were then made in water and 3 µL of each dilution was spotted on YPD plates that were exposed to approximately 25 J/m^2^ or no UV as a negative control. Plates were incubated at 30 °C for 2 days before pictures were taken.

### Reporting summary

Further information on research design is available in the Nature Portfolio Reporting Summary linked to this article.

## Supplementary information

Supplementary Information

Description of Additional Supplementary Files

Supplementary Data 1

Supplementary Data 2

Supplementary Data 3

Supplementary Data 4

Reporting Summary

## Data Availability

The Illumina sequencing reads generated in this study have been deposited in the NCBI short read archive database under accession code PRJNA876410 (for UV-treated *rad30*Δ yeast). Illumina reads for calling UV-induced mutations from WT and *rad26*Δ yeast were obtained from the NCBI short read archive database under accession code PRJNA605561. A complete list of mutations from whole genome sequencing used in this analyses is provided in Supplementary Information/ Supplementary Data 2. Coordinates for TTS and TES are available at https://hgdownload.soe.ucsc.edu/goldenPath/sacCer3/bigZips/genes. Origin of replication positions were obtained from http://cerevisiae.oridb.org/search.php?chr=all&confirmed=true&likely=true&dubious=true&name=.

## Code availability

Custom code is available on GitHub^62^ (https://github.com/S-RobertsLab/Vandenberg-et-al.−2023).
