# Supplementary material for: Contributions of replicative and translesion DNA polymerases to mutagenic bypass of canonical and atypical UV photoproducts
Source: Nat Commun. 2023 May 4;14:2576. doi: 10.1038/s41467-023-38255-5 (PMC10160025; doi:10.1038/s41467-023-38255-5)
Supplement: Supplementary file 1 — Supplementary Information [file 41467_2023_38255_MOESM1_ESM.pdf]

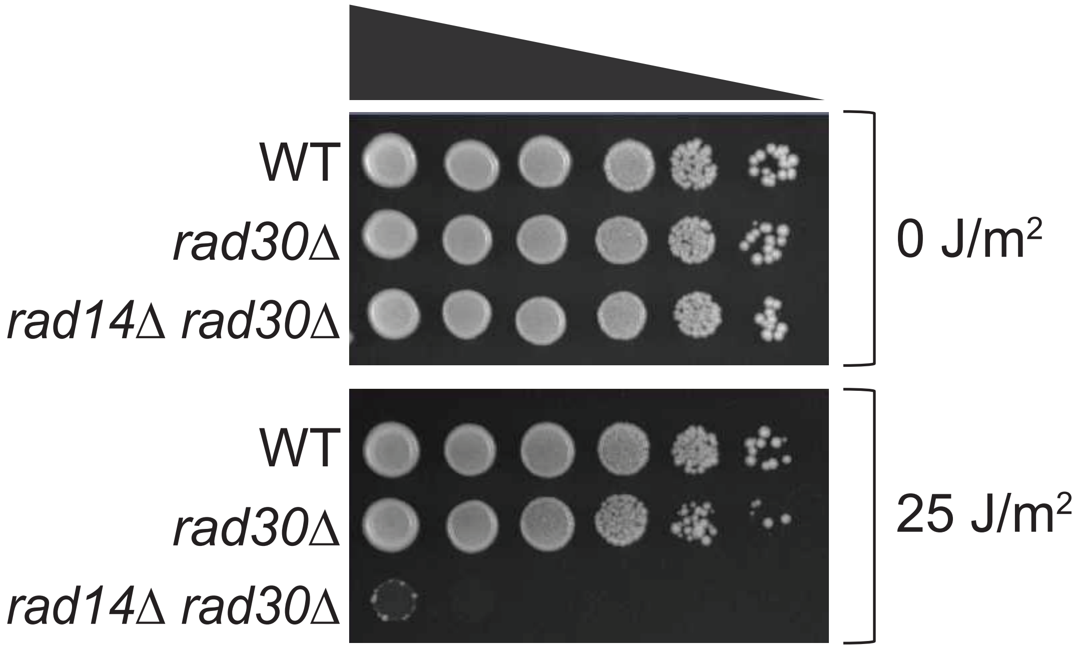

Supplementary Figure 1. UV Sensitivity of *rad30Δ rad14Δ* diploid yeast. Spot assay of decreasing concentrations of WT, *rad30Δ* and *rad30Δ rad14Δ* diploid yeast exposed to either no UVC (top) or 25 J/m2 UVC (bottom). Images were taken after 4 days of incubation at 30ºC.

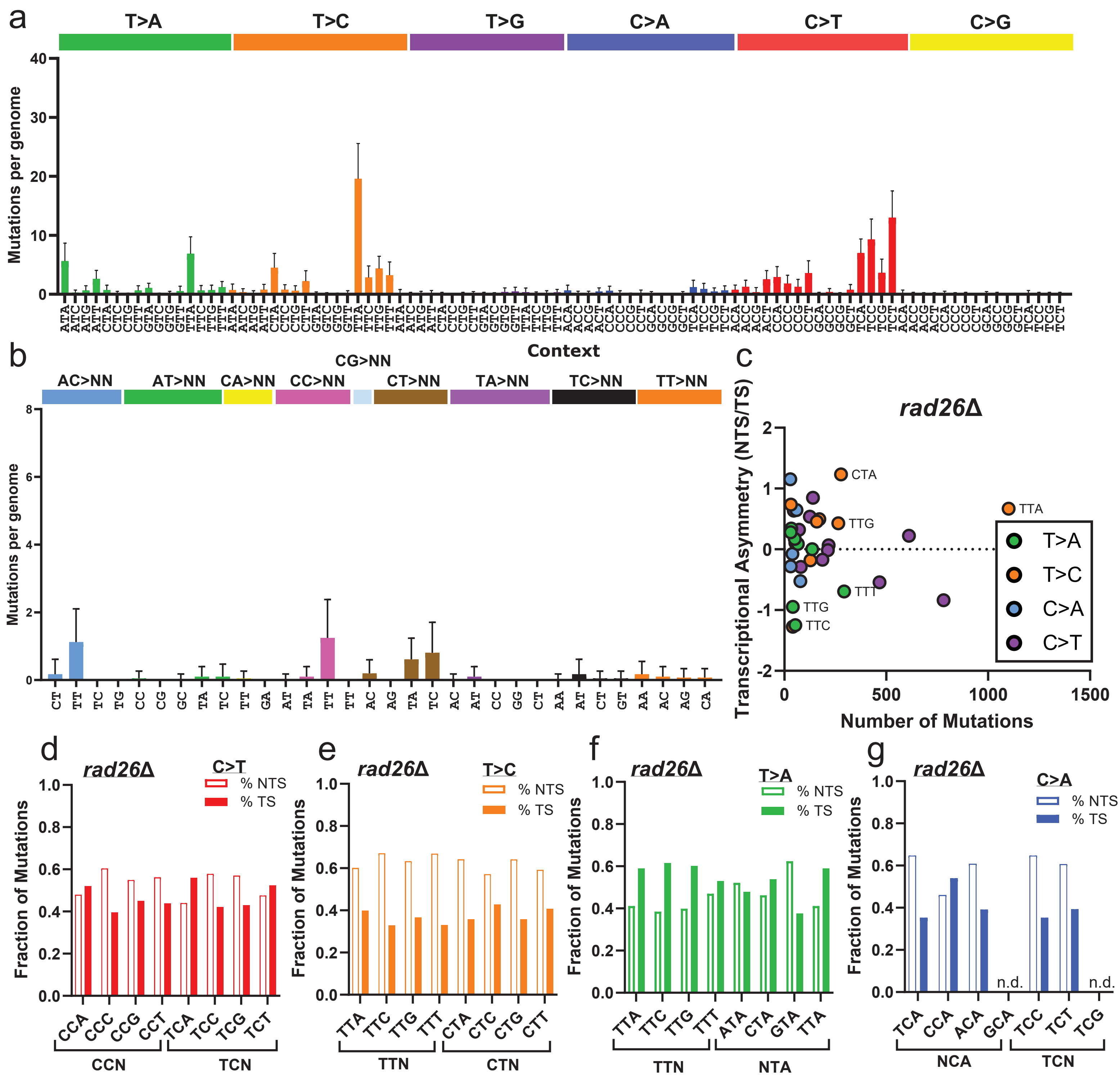

Supplementary Figure 2. Mutation Spectrum of rad26Δ yeast. (a) Mutation profile of single-nucleotide substitutions UVC-exposed *rad26Δ* yeast. 41 independent isolates were exposed to 25 J/m2 UVC (15x) and sequenced. Data are presented as mean values +/- SD for each substitution type (e.g., C>A, C>G, etc.) and trinucleotide context is depicted. Individual data points are not shown to maximize clarity. The middle base of each trinucleotide context is mutated. (b) Mutation profile of tandem nucleotide substitutions in 41 independent isolates of UVC-exposed *rad26Δ* yeast. The UV-induced mutation spectra of *rad26Δ* (n=41) and rad30Δ (n=49) yeast was compared by two-sided $\chi 2$ analysis. n= independent biological yeast isolates. (c) Transcriptional asymmetry (i.e., normalized ratio of mutations on NTS relative to TS across all yeast genes) is plotted relative to total number of mutations for each trinucleotide context in *rad26Δ* yeast. Only mutation classes with at least 30 mutations are plotted. The color of the circle indicates the mutation class (e.g., C>T). Statistical significance of transcriptional asymmetry in each mutation class and sequence context with at least 30 mutations was determined by a two-sided $\chi 2$ analysis using the expected number of mutations to be associated with the NTS and TS strands (Determined by calculating the frequency of trinucleotide contexts on each strand). The resulting p-values were corrected for multiple hypothesis testing using the Bonferroni method and listed in Supplementary Table 3. Fraction of (d) C>T, (e) T>C, (f) T>A, and (g) C>A mutations occurring on the non-transcribed strand relative to the transcribed strand for the indicated trinucleotide contexts in *rad26Δ* yeast. Bars for transcriptional asymmetry are representative of the percentage of total mutations occurring on either the NTS or TS for given substitution pair. As such, there is no variation. n.d. = not determined.

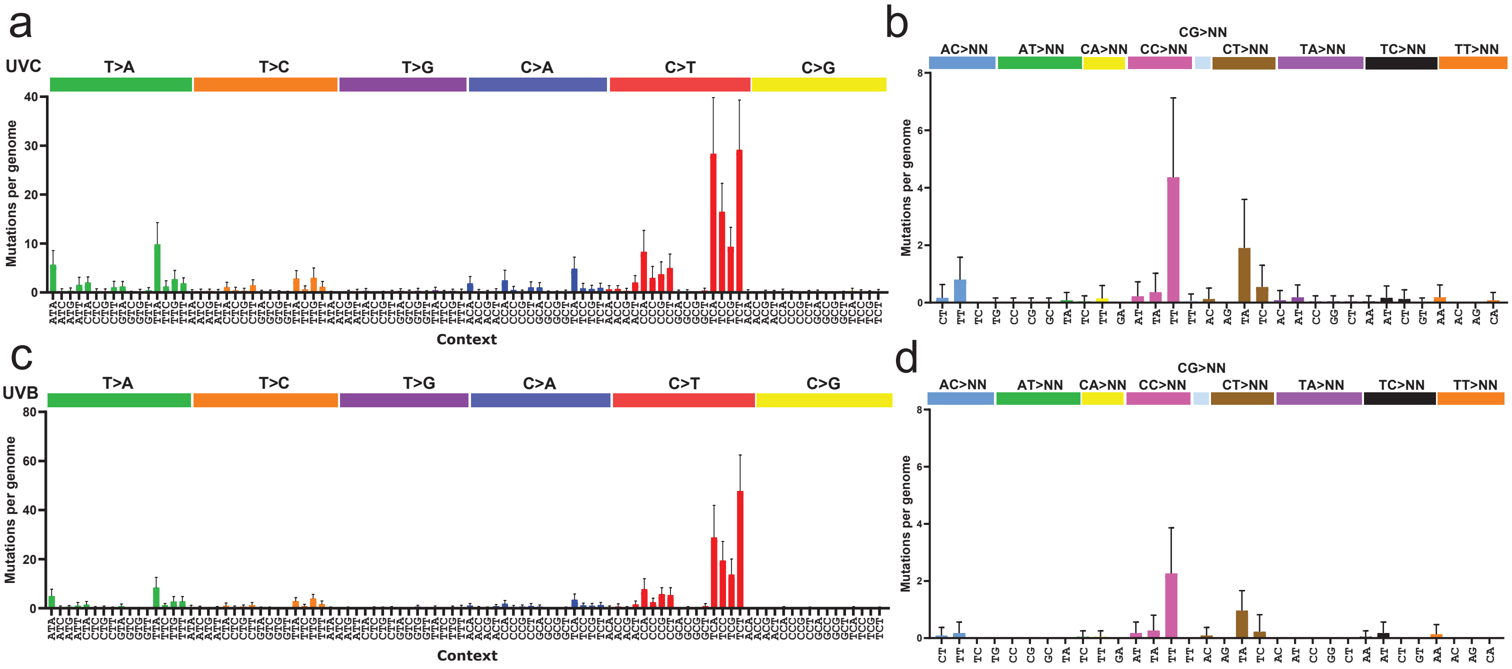

Supplementary Figure 3. Mutation spectra from whole genome sequencing of UVB irradiated *rad30*Δ yeast. (a) Data from Figure 2a. Data are presented as mean values +/- SD. (b) Data from Figure 2b. Data are presented as mean values +/- SD . (c) Mutation profile of single-nucleotide substitutions in UVB-exposed *rad30*Δ yeast. 22 independent isolates were sequenced. Data are presented as mean values +/- SD of the mutation count for each substitution type (e.g., C>A, C>G, etc.) and trinucleotide context is depicted. The middle base of each trinucleotide context is mutated. (d) Mutation profile of tandem nucleotide substitutions in 23 independent isolates of UVB-exposed *rad30*Δ yeast. UVB spectra show mutations from 29 (WT) and 23 (*rad30*Δ) independent isolates. Individual data points not shown to maximize clarity.

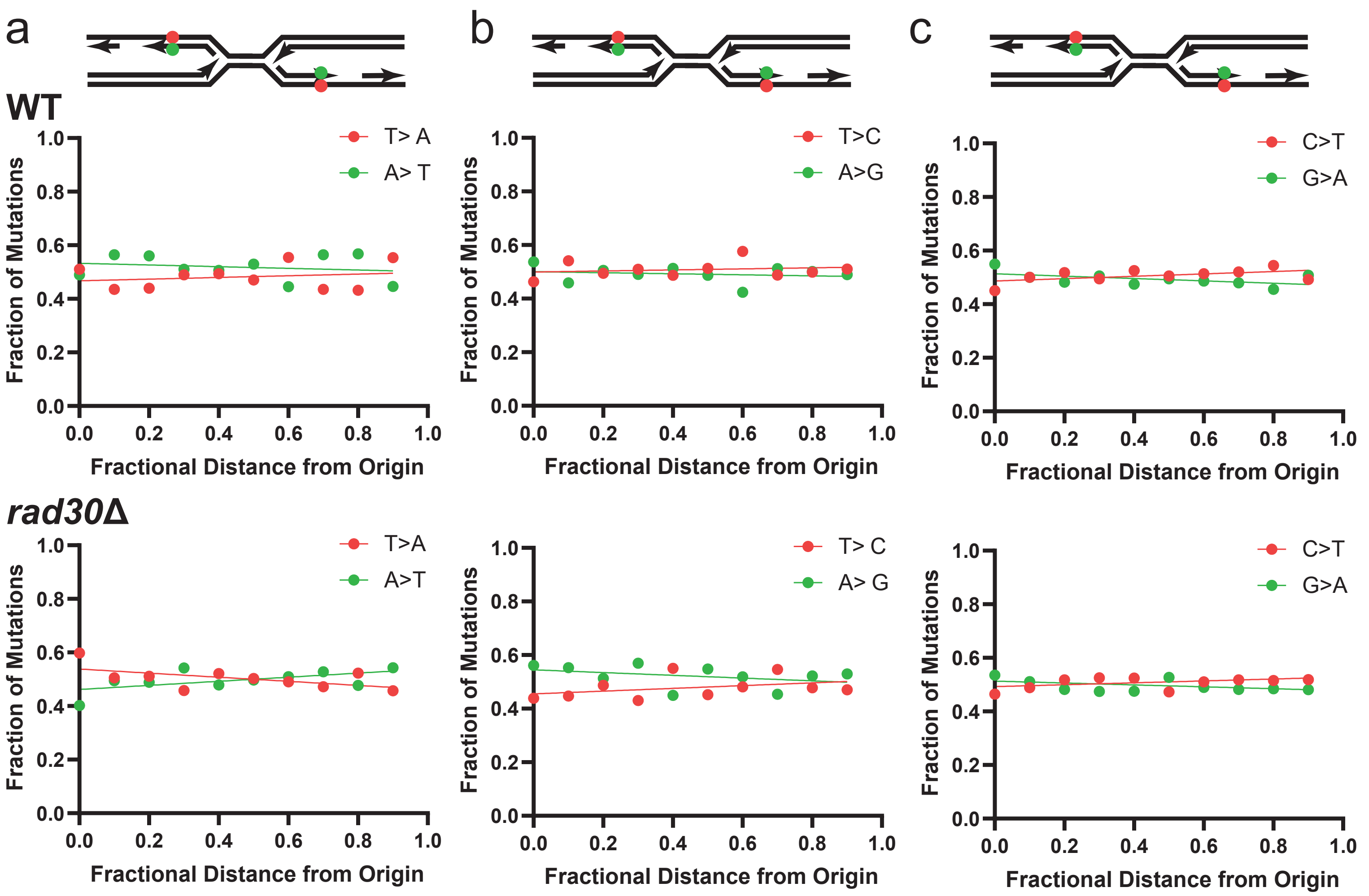

Supplementary Figure 4. Replication strand bias of UV-induced mutations in WT and *rad30Δ* yeast. (a) The relative abundance of UV-induced T>A mutations (red) and A>T mutations (green) in WT or *rad30Δ* yeast according to the fractional distance between neighboring replication origins. Lines represent linear trend lines fitting the fractional abundance of T>A (red) and A>T (green) mutations across the entire fractional distance between neighboring origins. Same as above, for (b) T>C (red) and A>G (green) and (c) C>T (red) and G>A (green) mutations.

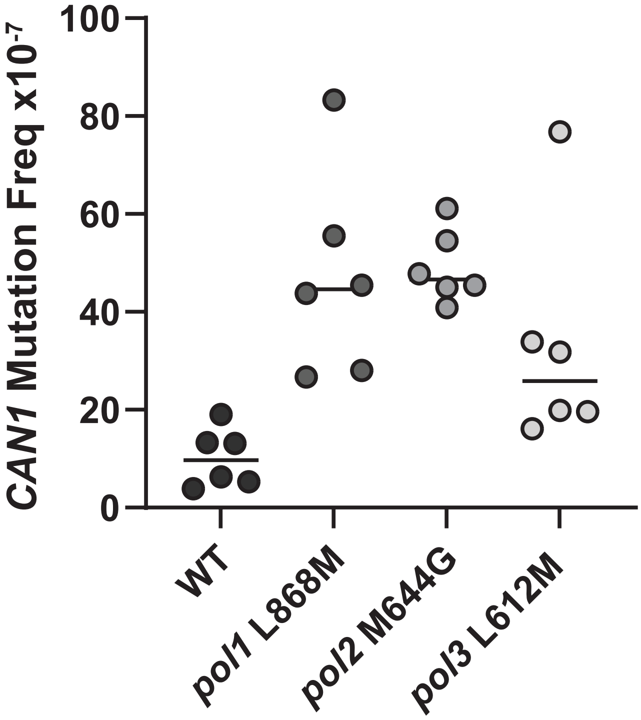

Supplementary Figure 5. Replicative polymerase mutants show expected mutator phenotypes. *CAN1* mutation assay of replicative polymerase mutants within yeast containing the *ura3* single-stranded reporter system. Median *CAN1* mutation frequency determined from six independent isolates per strain.
