# Supplementary material for: Contributions of replicative and translesion DNA polymerases to mutagenic bypass of canonical and atypical UV photoproducts
Source: Nat Commun. 2023 May 4;14:2576. doi: 10.1038/s41467-023-38255-5 (PMC10160025; doi:10.1038/s41467-023-38255-5)
Supplement: Supplementary file 2 — Description of Additional Supplementary Files [file 41467_2023_38255_MOESM2_ESM.pdf]

Description of Additional Supplementary Files

File Name: Supplementary Data 1
Description: Yeast strain list.

File Name: Supplementary Data 2
Description: Oligonucleotides used for yeast strain creation

File Name: Supplementary Data 3
Description: List of mutations identified by whole genome sequencing.

File Name: Supplementary Data 4
Description: P-values for $\chi 2$ tests in Figure 4 and Supplementary Figure 2.
