# Supplementary material for: Contributions of replicative and translesion DNA polymerases to mutagenic bypass of canonical and atypical UV photoproducts
Source: Nat Commun. 2023 May 4;14:2576. doi: 10.1038/s41467-023-38255-5 (PMC10160025; doi:10.1038/s41467-023-38255-5)
Supplement: Supplementary file 7 — Reporting Summary [file 41467_2023_38255_MOESM7_ESM.pdf]

nature portfolio

Corresponding author(s): Steven A. Roberts

Last updated by author(s): Mar 30, 2023

Reporting Summary

Nature Portfolio wishes to improve the reproducibility of the work that we publish. This form provides structure for consistency and transparency in reporting. For further information on Nature Portfolio policies, see our Editorial Policies and the Editorial Policy Checklist.

Statistics

For all statistical analyses, confirm that the following items are present in the figure legend, table legend, main text, or Methods section.

n/a	Confirmed	
☐	☒	The exact sample size (*n*) for each experimental group/condition, given as a discrete number and unit of measurement
☐	☒	A statement on whether measurements were taken from distinct samples or whether the same sample was measured repeatedly
☐	☒	The statistical test(s) used AND whether they are one- or two-sided<br>*Only common tests should be described solely by name; describe more complex techniques in the Methods section.*
☒	☐	A description of all covariates tested
☐	☒	A description of any assumptions or corrections, such as tests of normality and adjustment for multiple comparisons
☐	☒	A full description of the statistical parameters including central tendency (e.g. means) or other basic estimates (e.g. regression coefficient) AND variation (e.g. standard deviation) or associated estimates of uncertainty (e.g. confidence intervals)
☐	☒	For null hypothesis testing, the test statistic (e.g. *F*, *t*, *r*) with confidence intervals, effect sizes, degrees of freedom and *P* value noted<br>*Give P values as exact values whenever suitable.*
☒	☐	For Bayesian analysis, information on the choice of priors and Markov chain Monte Carlo settings
☒	☐	For hierarchical and complex designs, identification of the appropriate level for tests and full reporting of outcomes
☐	☒	Estimates of effect sizes (e.g. Cohen's *d*, Pearson's *r*), indicating how they were calculated

Our web collection on statistics for biologists contains articles on many of the points above.

Software and code

Policy information about availability of computer code

Data collection	not applicable
Data analysis	CLC genomics version 7.5 was used for sequence alignment and mutation calling. The further analysis used Bedtools v2.27.1 and custom RStudio 1.2.5033 or Python 3 scripts as provided on Github repository https://github.com/S-RobertsLab/Vandenberg-et-al.-2023. Statistical analyses not performed in RStudio or Python 3, were preformed with GraphPad Prism v6.07.

For manuscripts utilizing custom algorithms or software that are central to the research but not yet described in published literature, software must be made available to editors and reviewers. We strongly encourage code deposition in a community repository (e.g. GitHub). See the Nature Portfolio guidelines for submitting code & software for further information.

Data

Policy information about availability of data

All manuscripts must include a data availability statement. This statement should provide the following information, where applicable:

- Accession codes, unique identifiers, or web links for publicly available datasets
- A description of any restrictions on data availability
- For clinical datasets or third party data, please ensure that the statement adheres to our policy

The Illumina sequencing reads generated in this study have been deposited in the NCBI short read archive database under accession code PRJNA876410 (for UV-treated rad30Δ yeast). Illumina reads for calling UV-induced mutations from WT and rad26Δ yeast were obtained from the NCBI short read archive database under

accession code PRJNA605561. A complete list of mutations from whole genome sequencing used in this analyses is provided in the Supplementary Information/ Supplementary Table 2. Coordinates for TTS and TES are available at https://hgdownload.soe.ucsc.edu/goldenPath/sacCer3/bigZips/genes/. Origin of replication positions were obtained from http://cerevisiae.oridb.org/search.php?chr=all&confirmed=true&likely=true&dubious=true&name=.

Human research participants

Policy information about studies involving human research participants and Sex and Gender in Research.

Reporting on sex and gender	This study did not involve human research participants.
Population characteristics	This study did not involve human research participants.
Recruitment	This study did not involve human research participants.
Ethics oversight	This study did not involve human research participants.

Note that full information on the approval of the study protocol must also be provided in the manuscript.

Field-specific reporting

Please select the one below that is the best fit for your research. If you are not sure, read the appropriate sections before making your selection.

☒ Life sciences ☐ Behavioural & social sciences ☐ Ecological, evolutionary & environmental sciences

For a reference copy of the document with all sections, see nature.com/documents/nr-reporting-summary-flat.pdf

Life sciences study design

All studies must disclose on these points even when the disclosure is negative.

Sample size	No power calculations were used in this manuscript. Instead, the number of isolates for whole genome sequencing were chosen to maximize mutation counts, targeting approximately 10,000 mutations per genotype. This number of mutations provides a high enough mutation could to detect statistically significant differences in mutation rates between chromosome subfeatures by chi-square analysis. Reversion assays and CAN1 mutation assays utilized at least 6 independent measurements. 4 independent measurements is the minimum number of replicates sufficient to observe a statistically significant difference in these assays using nonparametric Mann-Whitney Ranked Sum tests. Increasing this value to 6 allows more accurate determination of the median value of the mutation frequency by minimizing the impact of outliers.
Data exclusions	No exclusions were made.
Replication	Multiple isolates were analyzed and evaluated for all experiments. Sequencing of UVC treated pol eta deficient strains were performed in two batches to eliminate batch sequencing biases. All reversion mutation and CAN1 mutation experiments report results from 6 independent replicate experiments. All attempts to replicate the experiments produced consistent results.
Randomization	No randomization was utilized. Yeast strains were separated into experimental groups by genotype. Covariates were controlled for by utilizing isogenic strains that only differ by the modification of a single gene. Multiple independently derived isolates for each experimental group were assessed in each experiment and produced comparable results, confirming that off-target changes to the genetics of yeast strains during construction was unlikely.
Blinding	No blinding was done because no exclusions were made and all measurements were objective.

Reporting for specific materials, systems and methods

We require information from authors about some types of materials, experimental systems and methods used in many studies. Here, indicate whether each material, system or method listed is relevant to your study. If you are not sure if a list item applies to your research, read the appropriate section before selecting a response.

Materials & experimental systems

n/a	Involved in the study
☒	☐ Antibodies
☒	☐ Eukaryotic cell lines
☒	☐ Palaeontology and archaeology
☒	☐ Animals and other organisms
☒	☐ Clinical data
☒	☐ Dual use research of concern

Methods

n/a	Involved in the study
☒	☐ ChIP-seq
☒	☐ Flow cytometry
☒	☐ MRI-based neuroimaging
